# Treatment of fifth metacarpal neck fractures with antegrade single elastic intramedullary nailing

**DOI:** 10.1186/s12891-017-1592-3

**Published:** 2017-06-02

**Authors:** Yuanshi She, Youjia Xu

**Affiliations:** 1 Department of Orthopedics, Nanjing Medical University Affiliated Suzhou Hospital (Suzhou Municipal Hospital), Daoqian Street No.26, Suzhou, Jiangsu 215002 China; 2 Department of Orthopedics, 2nd Affiliated Hospital of Soochow University, Sanxiang Road No.1055, Suzhou, Jiangsu 215000 China

**Keywords:** Metacarpal neck, Fracture, Antegrade single elastic nailing, Surgery

## Abstract

**Background:**

The aim of this study was to investigate clinical outcomes of fifth metacarpal neck fractures using antegrade single elastic nail and to explore ideal puncture point to avoid iatrogenic ulnar nerve injury.

**Methods:**

A single elastic nail with suitable diameter was used in 27 cases of fifth metacarpal neck fractures with dorsal angulation over 45°. An initial entry point was perforated at the ulnar-dorsal base of the metacarpal. The nail was inserted in an antegrade approach. The nail was usually removed at about 5 weeks postoperatively.

**Results:**

At final follow up, all fractures proceeded to bony union. The mean total passive motion was 285° and the mean total active motion (TAM) was 270°. The mean angulation decreased from 50.2 ± 6.3° preoperatively to 7.4 ± 2.3° postoperatively (*p* < 0.001). The mean DASH-Score was 2.1 ± 3.6 points after surgery. Two cases of skin irritation and one case of the dorsal cutaneous branch of the ulnar nerve (DCBUN) injury were observed. Superficial wound infections were not observed.

**Conclusions:**

Collectively, antegrade single elastic intramedullary nailing was a minimally invasive and reliable fixation technique for fifth metacarpal neck fractures with dorsal angulation over 45°. Appropriate puncture position helped to reduce nerve damage.

## Background

Fifth metacarpal neck fracture (known as boxer’s fractures) is the most common type of hand bone fractures. It amounts to 5% of all fractures in the upper extremity [1–3]. The fifth metacarpal neck fracture generally presented palmar angulation owing to the force of the interosseous muscles [4]. Unsuitable treatments may leave esthetic sequelae and metacarpophalangeal extension deficit. In the past, there were many controversies in the treatment of the fifth metacarpal neck fracture [5, 6]. Conservative treatment with reduction and immobilization was successful in most cases [7, 8]. However, a dorsal angulation less than 45 degrees can be treated conservatively [9]. A fracture angle greater than 45 degrees produces significant muscle shortening which can limit motion of the fifth digit, and surgery was usually indicated [10].

A range of procedures had been applied, including intermetacarpal K-wire [11], intramedullary K-wire [12] and locking plate [13]. However, each of them had some drawbacks clinically. K-wire fixed with an acute angle or entry point are very close to the fracture line, and this would lead to unstable fracture reduction. Plates may induced extensive soft tissue dissection, nonunion, and wound infections [14, 15]. Recently, elastic stable intramedullary nailing (ESIN) was also utilized for metacarpal fractures clinically with an excellent effect in children [14]. The advantages of ESIN include faster fracture healing, excellent functional and cosmetic results, safe and reliable surgical technique, and lower severe complication rate [15]. In addition, it was reported that antegrade intramedullary pinning has some clinical advantages during the early recovery period over percutaneous retrograde intramedullary pinning for the displaced fifth metacarpal neck fractures and antegrade intramedullary pinning can be recommended for patients who require an early return of hand function [10].

Therefore, the purpose of the present study was to retrospectively investigate the clinical outcomes using antegrade single elastic intramedullary nailing for fifth metacarpal fractures. The potential complications were also studied, including ulnar nerve injury. It was hypothesized that antegrade single elastic intramedullary nailing would make hand functional recovery and avoid complications for the treatment of fifth metacarpal fractures.

## Methods

### Participants

**Fig. 1:**
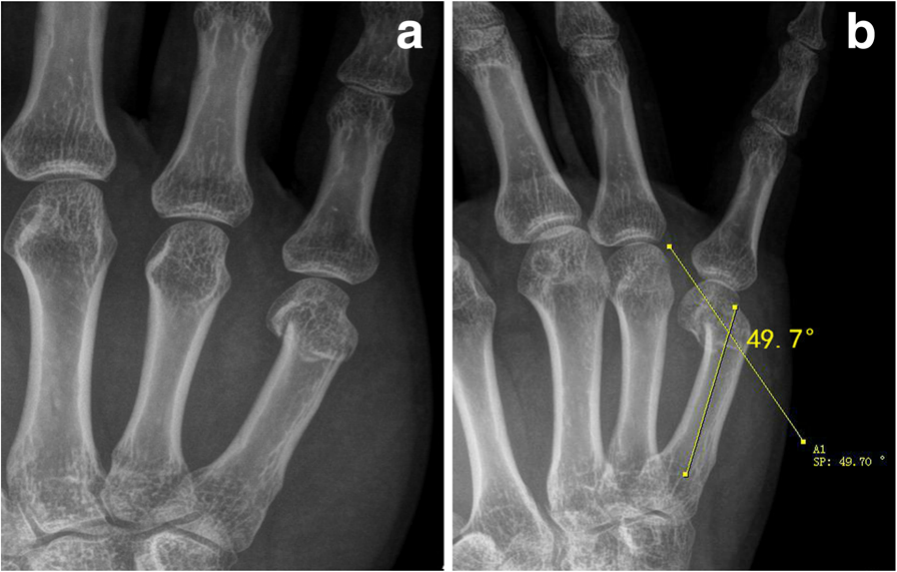
Typical fifth metacarpal neck fracture is shown preopepratively in the anteroposterior (**a**) and latero-oblique (**b**) planes with apex dorsal angulation 49.7degrees

This retrospective study was approved by the Health Sciences Institutional Review Board of our hospital, and written consent was obtained from all participants. An indication for the surgery was fifth metacarpal neck fractures (Fig. 1a, b) with apex dorsal angulation over 45°, with or without a rotational deformity. Exclusion criteria included: (1) severe comminuted fractures without unbroken metacarpal head, (2) time for visiting was more than 2 weeks after injury, (3) location infection, (4) rheumatoid arthritis, (5) gout.

### Surgical technique

**Fig. 2:**
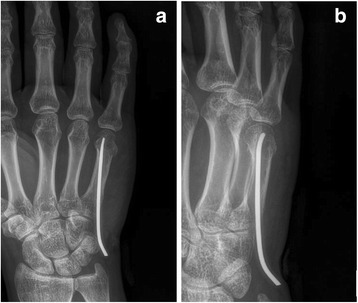
The fracture is treated with antegrade single ESIN achieving axial alignment in the anteroposterior (**a**) and latero-oblique (**b**) planes (with the distal of the nail dorsally)

First, the entry point was selected at the base of the ulnar dorsal border of the metacarpal using a needle under imaging guidance. Then, 3 mm skin incision was made close to the needle and subcutaneous tissue was bluntly separated to expose the bone. A small hole was made by a 2.5 mm drill. The distal side of the elastic nail was slightly bended. The operator inserted the nail in an antegrade approach through the hole. The fracture was reduced by manoeuvre and the nail passed across the fracture part (Fig. 2a, b).

### Follow-up

The information was accumulated from the admission records, operative records and Picture Archiving and Communication System (PACS). Patient demographics including age, sex, and other associated medical problems were collected. Operative data was collected regarding time to surgical intervention, operating time, intra-operative X-ray time, radiation exposure and diameter of the nail. Range of motion at the metacarpophalangeal (MCP) joint and inter phalangeal (IP) joint was assessed using TAM (total active motion) and TPM (total passive motion). Antero-posterior (AP) and latero-oblique X-rays were taken pre and post-operatively. Radiographs analyzed the fracture angulation. The complications were also documented.

## Results

Finally, twenty-seven fifth metacarpal neck fractures were investigated. The average age of the cohort was 23.6 years. The mean time from injury to surgical intervention was 6.7 days. The mean operation time was 19.5 min. Two types of elastic nails were used, including 1.5 mm diameter of nails (*n* = 12) and 2 mm diameter of nails (*n* = 15). Mean intra-operative X-ray time was 82 s and mean radiation exposure was 26.8 cGy/cm^2^. The mean correction angle of the metacarpal fracture was 42° (range 20 to 54) (Table 1).

**Table 1:** Patient demographic data

	Mean	Range
Follow-up time (weeks)	14.5	12–16
Gender	Male (*n* = 25), Female (*n* = 2)	
The time between injury and operation (days)	6.7	2–13
Operation time (min)	19.5	15–40
Elastic nail diameter	1.5 mm (*n* = 12), 2.0 mm (*n* = 15)	
Intra-operative X-ray time (sec)	82	76–102
Radiation exposure (cGy/cm^2^)	26.8	20–54
Fracture correction angle (°)	42	40–45

**Fig. 3:**
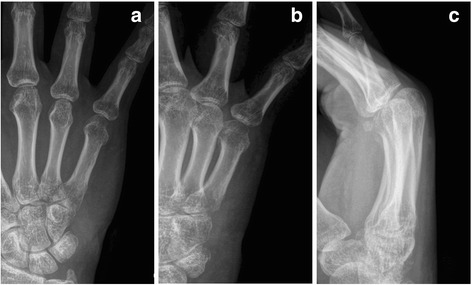
Status after the plant removal are shown (**a**-**c**).

Regarding the complications, two cases of skin irritation were observed. One case presented hypoesthesia of the dorsum of little finger, indicating dorsal cutaneous branch of ulnar nerve (DCBUN) injury. All of them recovered 3 months after surgery. Superficial wound infection was not observed. The fixation was removed at an average of 5.2 weeks if there was no tenderness and X-ray showed the fracture had bony union (Fig. 3a, b, c). All fractures proceeded to bony union at the follow-up time.

At the final follow-up, the mean total passive motion (TPM) was 285° (range 200°-330°) and the mean total active motion (TAM) was 270° (range 190°-315°). The mean angulation decreased from 50.2 ± 6.3° preoperatively to 7.4 ± 2.3° postoperatively (*p* < 0.001). The mean DASH-Score was 2.1 ± 3.6 points (Table 2).

**Table 2:** Patient Outcomes

	TPM(degree)	TAM(degree)	DASH Score	Angulation(degree)	
				Preoperative	Postoperative
Mean	285	270	2.1	50.2	7.4
SD	26	22	3.6	6.3	2.3
Range	200–330	190–315	1	47.3–68.5	6.5–10.2

*TPM* total passive motion, *TAM* total active motion, *DASH* Diasbilities of arm, shoulder and hand, *Angulation* apex dorsal angulation in lateral-oblique film

## Discussion

Previously, Marzouki et al. [16] presented a retrospective study of fifth metacarpal neck fractures with pins in an “L” configuration and obtained good clinical and radiological results. Potenza et al. [17] treated 28 patients with fifth metacarpal bone neck fractures using percutaneous transverse K-wire pinning techniqe, and reported that the surgical results were generally good at an average of 25 months after surgery. However, K-wires may lead to unstable fracture reduction and require auxiliary immobilization by splint after surgery [18]. In this study, single elastic nail by antegrade approach was used to treat metacarpal neck fracture. The single elastic nail acted on a three point intramedullary fixation providing adequate stability. Elastic stable intrumedullary nail had been established as the gold standard in the treatment of displaced and/or unstable diaphyseal fractures of the long bones and special metaphyseal fractures [19]. Collectively, the advantages of antegrade single elastic intramedullary nailing approach included (1) minimally invasive percutaneous techniques, (2) minimal trauma, (3) no affection on joint capsule, which is good for the joint function recovery, (4) accelerated fracture healing, (5) no influence on the extensor tendon.

In the present study, we employed single nail with oppropriate diameter in our operations. Compared with two or three nails, single nail not only simplified operative manipulation, alleviated trauma, but also provided adequate mechanical strength. Hiatt et al. [20] compared one 1.6-mm-diameter K-wire and three 0.8-mm-diameter K-wires of intramedullary fixation for transverse metacarpal shaft fractures and indicated that the increased stiffness of a single large-diameter construct provided more stability.

Previously, Kim et al. [10] reported that the mean DASH score of antegrade intramedullary pinning group (4.3) was smaller than that of the retrograde group (10.3) at 3 months after surgery. They suggested that antegrade intramedullary pinning had some clinical advantages during the early recovery period over retrograde intramedullary pinning for treatment of displaced fifth metacarpal neck fractures. Yammine et al. [6] investigated the outcomes of the antegrade intramedullary nailing (AIMN) compared to other surgical modalities in the treatment for fifth metacarpal neck fractures via a systematic review, and they summarized that that (a) AIMN demonstrated significantly better results in relation to grip strength at 12 months, TAM and ROM of the fifth finger; (b) AIMN technique yielded significantly lesser residual angulation at the site of fracture; (c) AIMN significantly demonstrated fewer complications; (d) there was a trend for better pain scores when using AIMN. Boussakri et al. [21] also reported that this minimally invasive percutaneous intramedullary nailing had good functional results and low morbidity in the treatment of metacarpal neck fractures. In the present study, antegrade approach was selected in all of our cases instead of retrograde approach because of the entry point far away from the fracture site. At final follow-up, it was observed that the mean TPM was 285°, the mean TAM was 270°, and the mean DASH-Score was 2.1 ± 3.6 points. The mean angulation decreased from 50.2 ± 6.3° preoperatively to 7.4 ± 2.3 ° postoperatively. Our present study was in accordance with these findings. It was reasonably believed that percutaneous single elastic intramedullary nailing for fifth metacarpal fractures was an excellent management.

One case of the DCBUN injury was presented after operation, and recovered 3 months later with oral Vit B12. DCBUN is one of the terminations of the ulnar nerve. This branch supplies sensation at the dorsoulnar aspect of the hand, the dorsum of the little finger, and the dorsoulnar aspect of the ring finger. Root et al. [22] reported that at least one longitudinal branch crossed dorsal to the extensor carpi ulnaris tendon prior to its insertion at the base of the fifth metacarpal in 82% specimens. It was realized that the longitudinal branch is vulnerable for traction, avulsion, or strangulation by operative manipulation. Therefore, we should make dissect incision and spread subcutaneous tissue bluntly avoiding iatrogenic injury instead of directly puncture.

## Conclusion

In conclusion, it was demonstrated that antegrade single intramedullary nailing was a minimally invasive and available procedure for boxers’ fractures, especially in cases with severe swelling or surrounding skin contusion. Antegrade single elastic intramedullary nailing would yield fast functional recovery of hand and avoid complications for the treatment of fifth metacarpal fractures.

## Abbreviations

AIMN: Antegrade intramedullary nailing
DCBUN: Dorsal cutaneous branch of the ulnar nerve
